# A simple, accurate and universal method for quantification of PCR

**DOI:** 10.1186/s12896-016-0256-y

**Published:** 2016-03-09

**Authors:** Nicky Boulter, Francia Garces Suarez, Stephen Schibeci, Trevor Sunderland, Ornella Tolhurst, Tegan Hunter, George Hodge, David Handelsman, Ulla Simanainen, Edward Hendriks, Karen Duggan

**Affiliations:** Accugen Pty Ltd, 11 Julius Avenue, North Ryde, NSW 2113 Australia; ANZAC Research Institute, University of Sydney, Sydney, NSW 2139 Australia; Westmead Millennium Institute, 176 Hawkesbury Road, Westmead, NSW 2145 Australia; Bitfuturistic Solutions, 9623 Lawndale Avenue SW, Lakewood, WA 98498 USA; Vectus Biosystems Pty Ltd, 11 Julius Avenue, North Ryde, NSW 2113 Australia

**Keywords:** qPCR, Absolute quantification, Universal calibrator, Gene expression

## Abstract

**Background:**

Research into gene expression enables scientists to decipher the complex regulatory networks that control fundamental biological processes. Quantitative real-time PCR (qPCR) is a powerful and ubiquitous method for interrogation of gene expression. Accurate quantification is essential for correct interpretation of qPCR data. However, conventional relative and absolute quantification methodologies often give erroneous results or are laborious to perform.

To overcome these failings, we developed an accurate, simple to use, universal calibrator, AccuCal.

**Results:**

Herein, we show that AccuCal quantification can be used with either dye- or probe-based detection methods and is accurate over a dynamic range of ≥10^5^ copies, for amplicons up to 500 base pairs (bp). By providing absolute quantification of *all* genes of interest, AccuCal exposes, and circumvents, the well-known biases of qPCR, thus allowing objective experimental conclusions to be drawn.

**Conclusion:**

We propose that AccuCal supersedes the traditional quantification methods of PCR.

**Electronic supplementary material:**

The online version of this article (doi:10.1186/s12896-016-0256-y) contains supplementary material, which is available to authorized users.

## Background

Significant differences in gene expression between tissues, disease states or treatment groups steer the direction of much research. It is imperative therefore, that mRNA quantification methods are standardized, accurate and unbiased. Due to its sensitivity, qPCR has become the standard method for measuring levels of gene expression. Quantification of PCR may be relative or absolute, and traditionally has been performed using non-specific intercalating dyes or gene-specific fluorescent probes. These methods, although widely used, are known to have many fundamental problems, despite considerable efforts over the last 20 years to overcome these.

Relative quantification using intercalating dyes is the most common method used. It is simple and cheap to perform, but relies on the use of one or more reference genes, against which the mRNA concentrations of the genes of interest (GOIs) are normalized [1]. The optimal number and choice of reference genes is determined empirically, but various useful computational methods help researchers in this regard [2, 3]. A suitable reference gene must be stably expressed between the experimental groups, have similar amplification efficiency and abundance to the GOIs. In reality this is rare, and reference genes often introduce bias into an experiment, leading to erroneous interpretation of results [4, 5].

Absolute quantification is performed by constructing a standard curve for each GOI and plotting the quantification cycle (Cq) values against log[quantity] of a dilution series of known GOI amount. These standards, comprising purified PCR product, plasmid DNA constructs or synthetic oligonucleotides spanning the PCR amplicon, are amplified, as are any experimental errors. This is important as the standard curve provides both the efficiency of the amplification primers and the amount of GOI in the unknown samples. Ideally, a new standard curve is generated each time a sample is quantified, but in practice, due to the complexity of the method, many researchers generate a standard curve once and use it repeatedly to quantify samples over a period of time. This produces inaccurate results as the efficiency of amplification may vary across samples, with time, or between the target used to generate the standard curve and the ‘real’ target within a complex sample [6].

The Minimum Information for publication of Quantitative real-time PCR Experiments (MIQE) guidelines [7] were introduced to facilitate standardization of the experimental and reporting practices in qPCR, to enable more reliable and unequivocal interpretation of qPCR results. These have helped enormously, but the fundamental problems associated with identifying suitable reference genes for an experiment, or having to generate standard curves for each GOI, are still present and can result in misinterpreted data.

AccuCal™, a universal *Accu*rate *Cal*ibrator, was developed to address these problems and aid in standardizing measurement in qPCR.

## Results

### Quantification using AccuCal

To address the long-standing problems of traditional PCR quantification methods [8], a methodological solution must integrate easily into each qPCR run, provide accurate results and ideally be universally applicable.

**Fig. 1:**
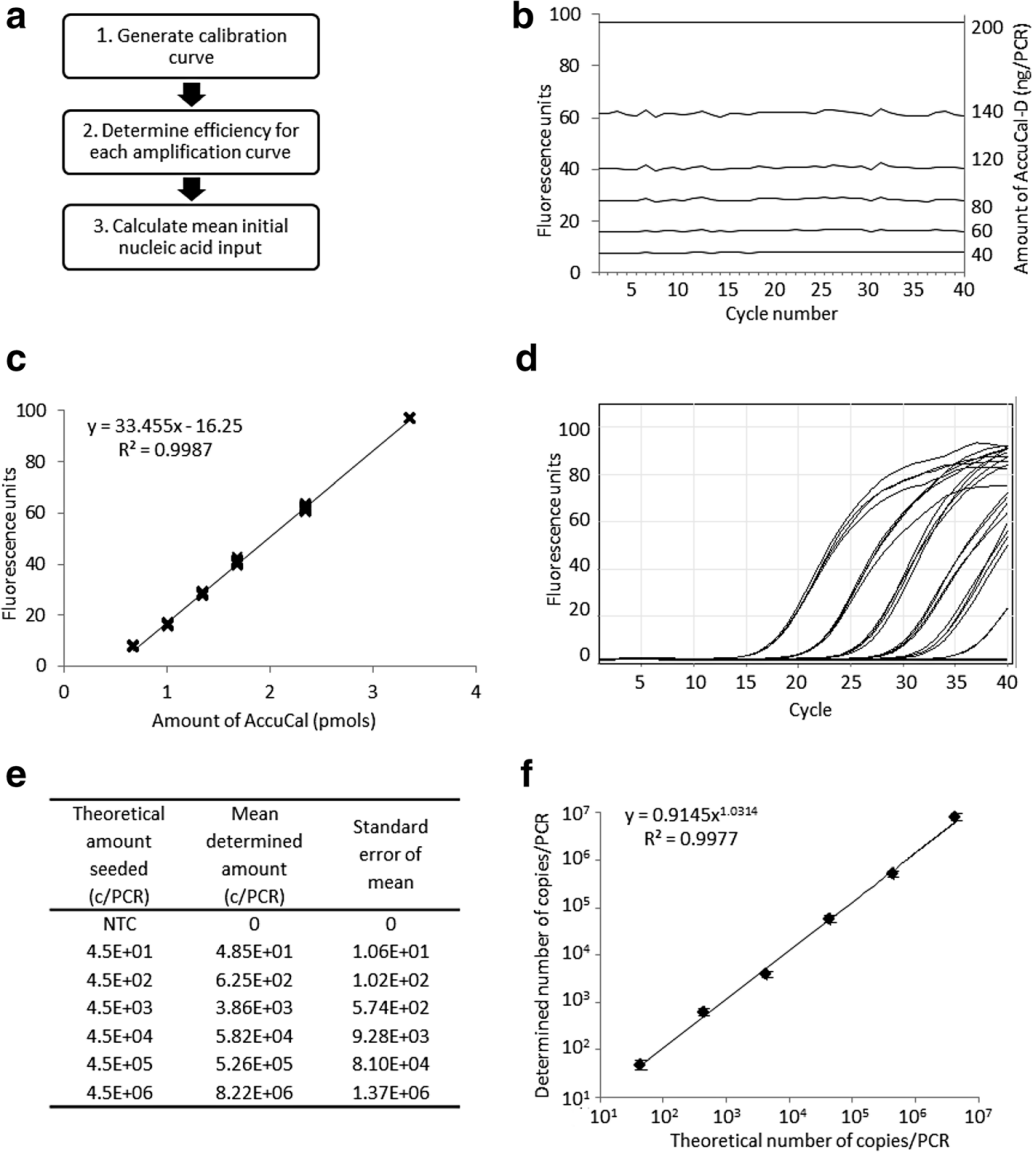
Quantification of PCR amplified nucleic acid using AccuCal calibrator. **a** The workflow associated with using AccuCal to quantify input nucleic acid amount in each PCR, **b** AccuCal calibrator was diluted so that 0, 40, 60, 80, 120, 140 and 200 ng in Sso Fast EvaGreen Supermix were added to respective wells of a PCR plate and subjected to 40 amplification cycles. **c** The fluorescence intensity of each AccuCal calibrator (after subtraction of mean 0 ng AccuCal fluorescence) was plotted against the amount (pmols) and a linear regression line fitted to generate the calibration curve. **d** Ten-fold dilutions of lambda DNA, ranging from 4.5 × 10^6^ – 4.5 × 10^1^ copies/PCR, were amplified in quadruplicate in Sso Fast EvaGreen Supermix on the same plate as the AccuCal calibrators. **e** The calibration curve was used, alongside the calculated efficiency of each amplification reaction and the cycle numbers between the take-off point (Cq) and second derivative maxima for each amplification reaction, to quantify the mean starting amount of DNA in each PCR. The standard error of the mean is also presented. **f** The theoretical and determined number of copies/PCR, plus SEM, were plotted against each other and a regression line drawn to demonstrate the agreement between the two values

The method relies on AccuCal-D, a double stranded DNA calibrator for use with intercalating dyes, or AccuCal-P, a single stranded, fluorescently labelled calibrator for probe-based qPCR assays. An initial optimization is required, but following this, the AccuCal method involves three simple steps (Fig. 1a and detailed in the Methods). RealCount™ software was developed to automate the computational steps.

To determine the AccuCal-D range to use, the initial optimization was performed under the same reaction conditions as for DNA amplifications. In the example shown, a range of 0–140 ng, was optimal, as this spanned the exponential portion of the amplification curves, where the amount of amplified target is directly proportional to the input amount [9, 10], and gave a linear calibration curve with R^2^ value of 0.9987 (Fig. 1b, c and Additional file 1). This determination only needs to be performed once, provided reaction conditions of all subsequent PCRs remain constant.

To show the quantification accuracy of AccuCal-D, we amplified serial ten-fold dilutions of known quantities of a 92 bp amplicon from lambda DNA, from 4.5 × 10^6^ – 4.5 × 10^1^ copies, in quadruplicate alongside AccuCal-D, at the predetermined amounts, and plotted the calibration curve (Fig. 1b-d). The efficiency of each amplification reaction was then determined by RealCount using known algorithms [10]. Finally, using the efficiency values and calibration curve, the mean amount of input DNA, and standard error of the mean, was calculated for all cycles during the exponential phase of each amplification curve using RealCount (Fig. 1e). A regression analysis between the determined values and the theoretical amount seeded into the PCR yielded an R^2^ of 0.9977 (Fig. 1f) demonstrating the utility of the AccuCal-D method and its accuracy in absolute quantification of real-time qPCR.

**Fig. 2:**
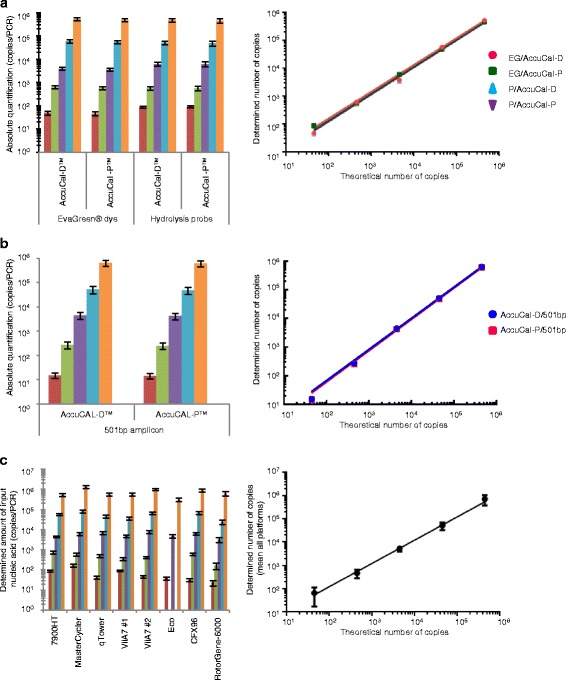
Quantification using both AccuCal-D and AccuCal-P. **a** Five, ten-fold dilutions of a known quantity of lambda DNA, ranging from 4.5 × 10^5^ to 4.5 × 10^1^, were amplified twice in quadruplicate and detected using either EvaGreen, the intercalating dye in Sso Fast mastermix, or a FAM-labelled hydrolysis probe specific to the target amplicon. In both cases, AccuCal-D and AccuCal-P calibrators were included on the same plate and used to independently quantify the starting amount of input DNA in each PCR. The theoretical amount versus the calculated amount, determined by either AccuCal-D or AccuCal-P, using the EvaGreen dye (EG) or the hydrolysis probe (P), was plotted and the linear regression of each is shown in the graph on the right. **b** Five, ten-fold dilutions of a known quantity of lambda DNA in quadruplicate were amplified in Sso Fast mastermix using primers to give a 501 bp amplicon. AccuCal-D and AccuCal-P calibrators were included on the same plate and were used to independently quantify the starting amount of lambda DNA in each PCR. The theoretical amount versus the calculated amount, determined by either AccuCal-D or AccuCal-P, for the 501 bp amplicon, was plotted and the linear regression of each is shown in the graph on the right. **c** Five, ten-fold dilutions (3 one-hundred fold dilutions on the Eco) of a known quantity of lambda DNA were amplified in various mastermixes (see Methods) on the different qPCR platforms indicated over 2–10 PCR runs. The theoretical amount versus the mean calculated amount, determined by AccuCal-D, across all platforms was plotted and the linear regression is shown in the graph on the right. The mean number of calculated copies/PCR and SEM are shown in each case

AccuCal-D relies on an intercalating dye to generate fluorescence, but the dye:AccuCal-D fluorescence ratio is unknown. To understand this relationship, we developed a probe-based version of AccuCal, AccuCal-P. A 92 bp amplicon from a range of concentrations of lambda DNA was amplified and detected using either a FAM-labelled hydrolysis probe or EvaGreen intercalating dye. Both AccuCal-D and FAM-labelled AccuCal-P were included on the PCR plate and were used independently to quantify the lambda DNA detected by both markers. The quantification using either AccuCal-D or AccuCal-P, for both sets of PCR amplifications, yielded indistinguishable results for each dilution with no significant differences between the slopes when theoretical number of copies is plotted against determined number of copies (slopes = 0.9601, 0.9653, 0.9623 and 0.9701, R^2^ = 1 for each; Fig. 2a and Additional file 1). AccuCal-P and the hydrolysis probe are labelled with one FAM moiety per DNA molecule, and report the same fluorescence per DNA molecule as the EvaGreen dye does under these qPCR conditions.

To determine the range of amplicon sizes for which AccuCal-D quantification can be used, we also amplified lambda amplicons of 501 bp. Amplicons of 92 bp to 501 bp covers the spectrum of amplicon sizes that are typically amplified by qPCR. Again, AccuCal-P and a FAM-labelled template-specific hydrolysis probe were used as a comparator for AccuCal-D and intercalating dye. The results show that the quantification is similar for both amplicon sizes whether this is calculated using AccuCal-D or AccuCal-P, with neither slope differing significantly from 1 (Fig. 2b). This suggests that the dye and probe fluorescence remains constant over this range of amplicon sizes and therefore AccuCal can be reliably used to quantify any amplicon within this range.

To evaluate the performance of AccuCal-D in a variety of dye-based mastermixes on a number of real-time qPCR platforms, eight independent research groups were provided with AccuCal-D and reagents for lambda amplification (92 bp amplicon). Each laboratory amplified their GOIs and known input amounts of lambda under a range of conditions typical for those laboratories. The results show that AccuCal-D provides an accurate, absolute quantification of known concentrations of lambda DNA in these varied and independent tests (Fig. 2c). When compared collectively across all platforms, the mean determined quantification correlates perfectly with the theoretical number of copies in each PCR (Fig. 2c, slope = 1).

When integrated onto each qPCR run under the same conditions as the GOI(s), AccuCal provides robust absolute quantification over a range of input amounts and amplicon sizes, in dye- or probe-based assays.

### AccuCal provides confidence in relative quantification analysis

Relative quantification e.g. ΔΔCq [11] and Pfaffl [12] analyses, has traditionally been the simplest and most commonly used method of PCR quantification. Although AccuCal provides absolute quantification, it can be applied relatively.

**Fig. 3:**
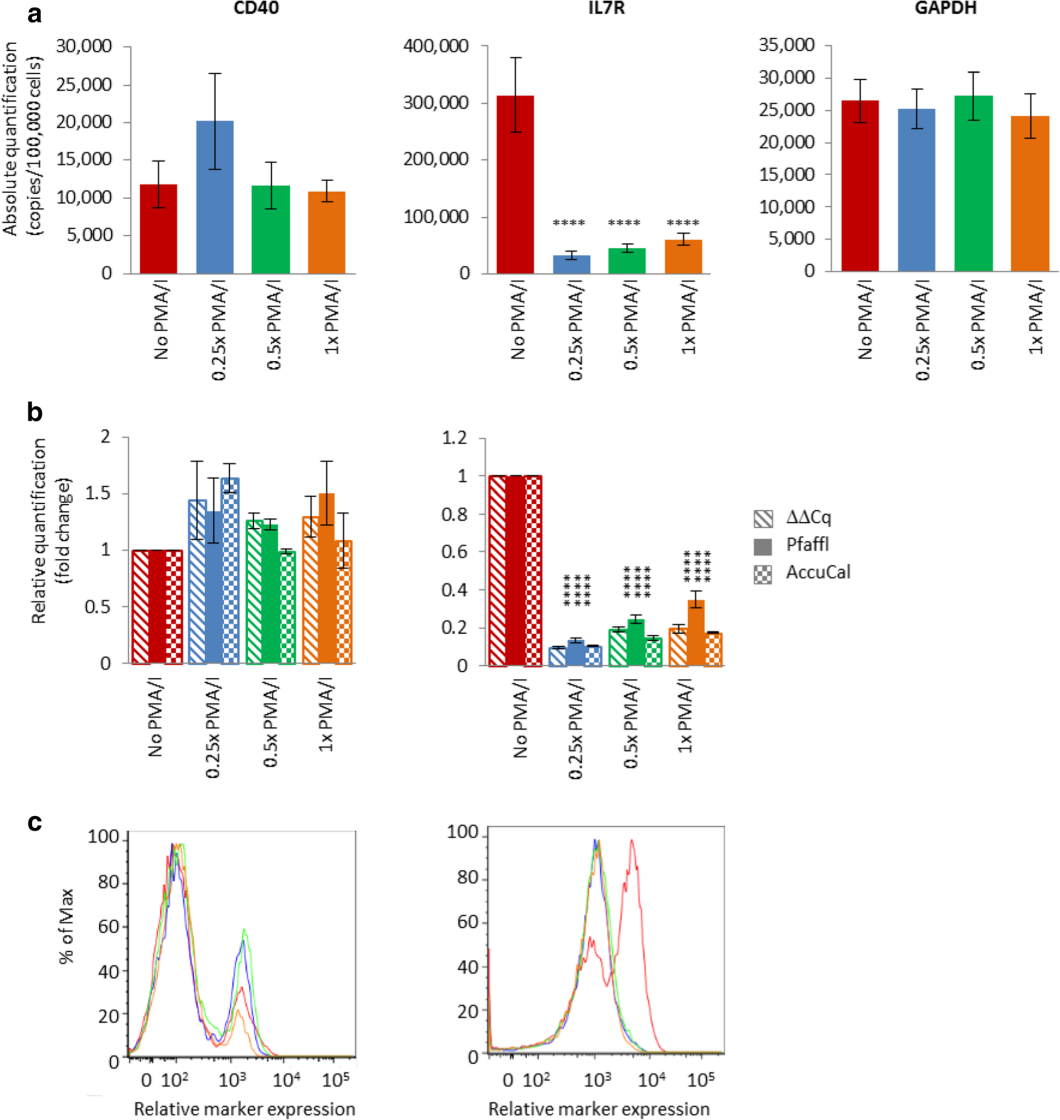
Quantification of *CD40*, *IL7R* and *GAPDH* in PBMCs stimulated with 0–1x PMA/ionomycin. **a** Absolute quantification of *CD40*, *IL7R* and *GAPDH* in PBMCs stimulated with 0, 0.25x, 0.5x and 1x PMA/ionomycin (20 ng ml^−1^ PMA, 500 ng ml^−1^ ionomycin; PMA/I) by RealCount software following qPCR using AccuCal-D calibrators. **b** Relative expression levels of *CD40* and *IL7R* in PBMCs stimulated with 0, 0.25x, 0.5x and 1x PMA/ionomycin (20 ng ml^−1^ PMA, 500 ng ml^−1^ ionomycin). The hatched bars are relative expression levels determined by ΔΔCq using *GAPDH* as the reference gene and no PMA/ionomycin as the control sample, solid bars are relative expression levels determined by Pfaffl analysis, using GAPDH as reference gene, unstimulated cells as controls and individual efficiency values calculated by RealCount software, and the checkered bars are quantified by RealCount software following inclusion of AccuCal-D in the same PCR run, and expressed relative to the no PMA/ionomycin control. **c** Representative overlay graphs from flow cytometry showing relative measurement of CD40 and IL7R in the same population of PBMCs stimulated with 0 (*red*), 0.25x (*blue*), 0.5x (*green*) and 1x PMA/ionomycin (20 ng ml^−1^ PMA, 500 ng ml^−1^ ionomycin; *orange*) as in (**a**). **** *p* < 0.0001 relative to respective no PMA/ionomycin control, *n* = 4

To compare AccuCal-D with ΔΔCq and Pfaffl analyses, we assessed levels of CD40 and Interleukin 7 receptor alpha chain (IL7R) variants. Activation of human peripheral blood mononuclear cells (PBMCs) reveals a repertoire of splice variants of these genes which reflect a predisposition to multiple sclerosis [13, 14]. We conducted experiments to measure levels of *CD40* and *IL7R* in human PBMCs via qPCR following 24 h activation with varying amounts of phorbol myristate acetate (PMA) and ionomycin (PMA/I). Absolute quantification of the qPCR was performed using AccuCal-D and RealCount (Fig. 3a and Additional file 1). Relative quantification was assessed by expressing the absolute AccuCal-D values relative to the no PMA/ionomycin control, or by traditional ΔΔCq or Pfaffl analyses using glyceraldehyde 3-phosphate dehydrogenase (*GAPDH*) as a reference gene and the unstimulated cells as a control (Fig. 3b). For Pfaffl analysis, the efficiencies calculated by RealCount were used.

Both the absolute and relative analyses showed the expression of *IL7R* was 3–10 fold lower in stimulated cells (*p* ≤ 0.0001 by all methods) compared to unstimulated cells whereas any differences in the levels of *CD40* were of no great significance. In this experiment, the interpretation of the qPCR data from the ΔΔCq and Pfaffl analyses was the same as that provided by AccuCal-D (Fig. 3b). The assumption for ΔΔCq and Pfaffl analyses is that the level of *GAPDH* reference gene remains constant between treatments. Importantly, absolute quantification using AccuCal-D indicated that this was indeed the case (Fig. 3a). The results of the qPCR analyses were supported by flow cytometry, showing no difference in the level of CD40 expression and a 3–5.5 fold decrease in expression of *IL7R* in the treatment group compared to the untreated cells (Fig. 3c).

Importantly, AccuCal-D and RealCount analysis provides data regarding the expression levels of all genes, including the reference gene, between treatments/groups (Fig. 3a), and the individual efficiencies for each amplification reaction, which are not available using ΔΔCq and Pfaffl analyses.

### AccuCal supersedes traditional quantification analyses

**Fig. 4:**
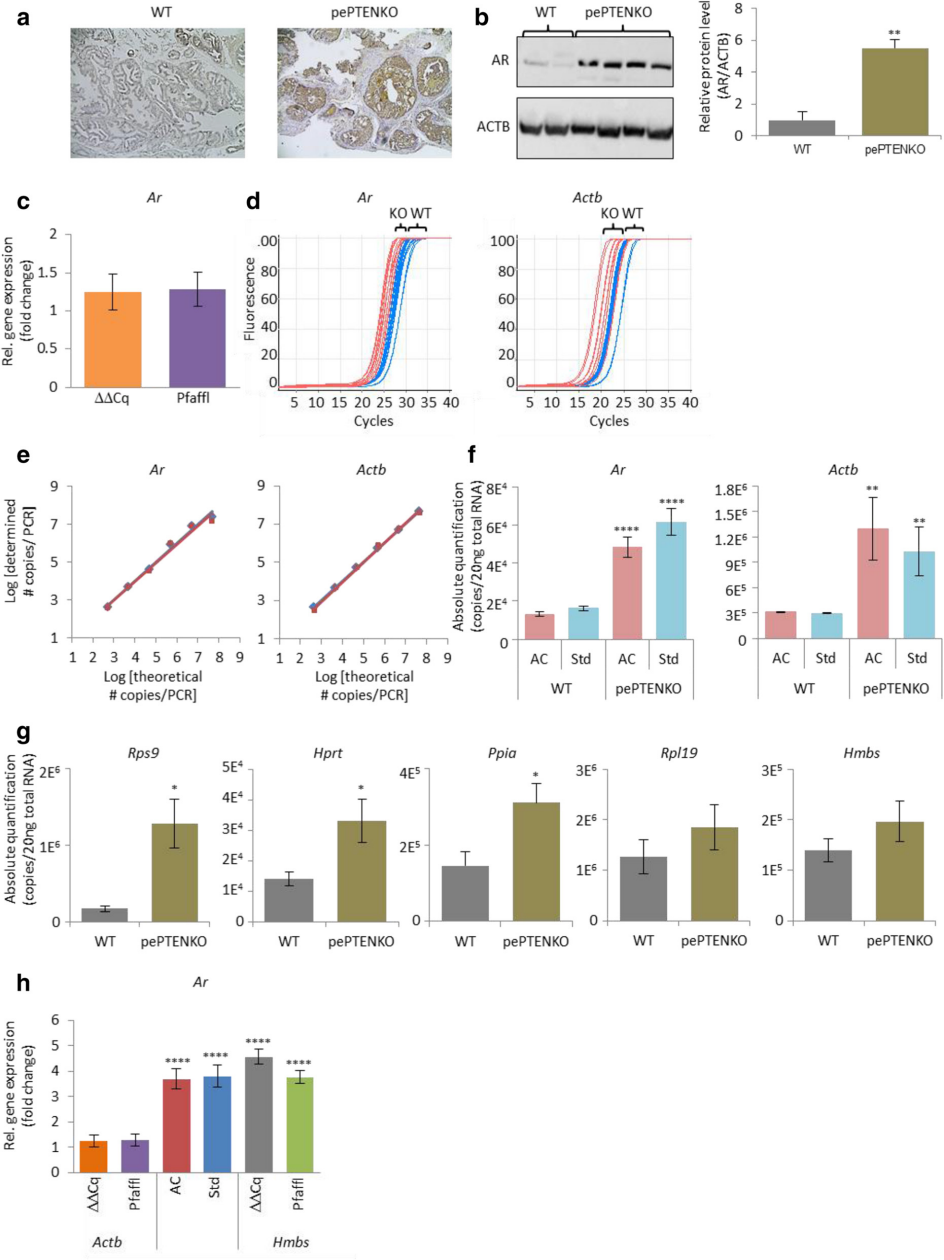
Quantification of protein and mRNA levels of androgen receptor (AR, *Ar*) in mouse anterior prostate. **a** Representative immunohistochemistry showing AR protein expression (brown staining) and differential pathology in prostate of WT and pePTENKO mice. **b** Quantification of AR protein by Western blot in anterior prostates of WT (*n* = 2) and pePTENKO (*n* = 4) mice, using β-actin (ACTB) as a loading control to determine relative protein levels. **c** Relative quantification of *Ar* by ΔΔCq and Pfaffl analyses using *Actb* as the reference gene and WT as the control. **d** qPCR of *Ar* and *Actb* in WT (*blue curves*, *n* = 7 in duplicate) and pePTENKO (*red curves*, *n* = 5 in duplicate) mice. **e** Comparison of theoretical versus determined quantification of serial dilutions of plasmids containing *Ar* or *Actb* amplicons, by either traditional standard curves (*blue diamonds and line*) or use of AccuCal and RealCount (*red squares and line*). **f** Absolute mRNA copy number quantification of *Ar* and *Actb* reference gene in the anterior prostate of WT (*n* = 7) and pePTENKO (*n* = 5) mice as determined by RT-qPCR with AccuCal calibrators and RealCount software (AC) or standard curves (Std). **g** Absolute quantification of a number of reference genes using AccuCal and RealCount for both WT (*n* = 7) and pePTENKO (*n* = 5) mice. **h** Comparison of relative quantification methodologies for *Ar.* ΔΔCq and Pfaffl analyses used either *Actb* or *Hmbs* as the reference gene and WT as the control, and AccuCal (AC) and standard curve (Std) absolute values were expressed in a relative manner to WT as the control. All graphed data is displayed as mean ± SEM. **** p <0.0001, ** *p* = 0.001–0.01, * *p* = 0.01–0.05 by independent two sample *t*-test between expression of androgen receptor or β-actin mRNA or protein in pePTENKO mice compared to WT mice, or between relative expression methods compared with ΔΔCq and Pfaffl performed using *Actb* as reference gene

Prostate epithelium-specific phosphatase and tensin homolog knockout (pePTENKO) induces prostate pathology [15] and modifies prostate specific androgen receptor (AR) expression in mice as determined by immunohistochemistry (Fig. 4a and Additional file 1) or Western blot (Fig. 4b). The Western analysis showed that levels of β-actin (ACTB) protein were constant and were used to determine relative protein expression levels. The AR protein content was significantly greater (*p* = 0.008) in prostate tissue from pePTENKO mice compared to wild-type (WT; Fig. 4b).

To determine whether these complex assays could be replaced by mRNA analysis using qPCR, we PCR amplified *Ar* and *Actb* from prostate RNA extracted from WT and pePTENKO mice. Initially, relative quantification was undertaken by traditional ΔΔCq and Pfaffl analyses with *Actb* as the reference gene and WT as the control. *Actb* was chosen as the protein was stably expressed between groups (Fig. 4b). The ΔΔCq and Pfaffl analyses indicated that there was no significant change in *Ar* expression levels in pePTENKO mice compared to WT (1.250 and 1.286 fold increase, respectively; Fig. 4c). It is known that protein and mRNA levels do not necessarily correlate [16, 17], which may explain this result. Alternatively, the relative qPCR analysis may be incorrect. Examination of the amplification plots suggested a greater expression of *Ar*, as well as *Actb,* in the prostate of pePTENKO mice (Fig. 4d).

To resolve this issue, we used absolute quantification via AccuCal-D, or standard curves, to determine the levels of *Ar* and *Actb* in pePTENKO and WT mice. The results demonstrated that expression levels of each gene had similar absolute quantifications by both methods (Fig. 4e) and were significantly higher in prostate from pePTENKO mice than WT mice for both standard curve and AccuCal-D (*Ar, p* < 0.0001 and *p* < 0.0001; *Actb*, *p* = 0.0049 and *p* = 0.0043, respectively; Fig. 4f). When expressed in a relative manner, *Ar* expression in pePTENKO mice was 3.806 fold higher than WT mice by standard curve quantification and 3.697 fold higher by AccuCal-D quantification. Both of these were significantly different from the ΔΔCq and Pfaffl analyses using *Actb* as a reference gene (*p* < 0.0001; Fig. 4h), but were in accord with the Western blot results. In this example, the ΔΔCq and Pfaffl analyses were not accurate because the reference gene used changed similarly to the GOI between the phenotypes (Fig. 4f).

Additionally, we quantified, with AccuCal-D, a number of other possible reference genes to use for normalization. There was no significant difference between the expression of ribosomal protein L19 (*Rpl19*) and hydroxymethylbilane synthase (*Hmbs*) between WT and pePTENKO, but the remaining reference genes were all expressed significantly more abundantly in the prostates of pePTENKO mice than WT mice, highlighting the difficulty in finding a suitable reference gene in some experiments (Fig. 4g). We then repeated the ΔΔCq and Pfaffl analyses using the least variable reference gene, *Hmbs*, and the results (4.294 and 3.603 fold increases, respectively) were very similar to those obtained using AccuCal-D or standard curve quantification, and significantly different from the ΔΔCq and Pfaffl analyses undertaken using *Actb* as a reference gene (*p* < 0.0001 in both cases; Fig. 4h).

In the exemplar described, AccuCal-D provided an alternative quantification method, independent of reference genes, where these are difficult to find (Fig. 4h). Importantly, we have also shown that absolute quantification by standard curve perfectly matched AccuCal-D analysis (Fig. 4e, f). This demonstrated that the protein and gene expression levels of AR/*Ar* correlated, allowing the use of mRNA analysis by qPCR as a faithful reporter assay. Notably, AccuCal provided this information much more simply than the standard curve method, and a single calibration curve could be used to quantify all genes investigated concurrently.

## Discussion

Traditional relative and absolute methods of qPCR have many, well-accepted flaws and errors [7]. The MIQE guidelines sought to minimize these, but the problems associated with reference genes, and the need to construct a standard curve for each GOI, remain. To overcome these limitations, an accurate, universal calibrator (AccuCal) was developed.

AccuCal can be included in any dye- (AccuCal-D) or probe-based (AccuCal-P) qPCR experiment. The technology works robustly for amplicons up to 500 bp in length over a dynamic range of ≥10^5^ copies, and in a range of mastermixes, real-time PCR platforms and laboratories. A range of AccuCal concentrations is included in each qPCR run and used to generate a calibration curve to quantify the amount of any GOI. Significantly, AccuCal fluoresces proportionally to the amount of DNA in the well but is not PCR amplified, thus minimizing errors in quantification (e.g. due to errors in pipetting or spectrophotometric estimation of input nucleic acid concentrations) [18]. Customized software, RealCount, automates the quantification calculations, streamlining data analysis.

Essential to current relative quantification methods are reference genes and the assumption that these do not change between treatments or groups. Many studies have shown that commonly used reference genes vary with experimental conditions and between tissues, while some, such as *Actb* and *GAPDH*, have pseudogenes [19] which may produce specific amplification products in an mRNA-independent fashion [20]. The MIQE guidelines recommend the use of multiple reference genes to provide a more accurate normalization [7]. However, in some experiments, this can be very difficult to achieve, as exemplified in our mouse prostate example. AccuCal provides quantification independently of reference genes and is therefore a much simpler method to use and is devoid of these problems.

Reference genes are also often included in gene expression studies to account for differences in reverse transcription (RT) efficiencies between experiments. These have been shown to often result in relative gene differences of 2–5 fold or more [21, 22] depending upon the RNA concentration and integrity, the RT enzyme used, the priming strategy employed, the sample used and reaction conditions [23]. RT differences have also been shown to be gene dependent which questions the validity of using any gene as a normalizer for RT and PCR and may mask results, leading to erroneous interpretation of data. Methods that help resolve differences in RT efficiency are required, but in their absence, differences in gene expression <5 fold need to be construed cautiously.

AccuCal allows the researcher to objectively see any differences in absolute gene expression of *all* genes. This potentially unmasks differences in RT efficiency and provides valuable information regarding reference gene expression, giving confidence, or otherwise, to the researcher about reference gene choice or RT differences. In cases where reference genes are stable, the results generated by AccuCal and traditional relative methods agree, but when the reference gene expression is not stable between samples, relative quantification leads to incorrect interpretation, whereas AccuCal provides an unbiased, interpretable result.

The current gold standard in qPCR is absolute quantification using standard curves. Comparison of the quantification of *Ar* and *Actb* by AccuCal or standard curves demonstrated that the results were completely interchangeable. The major impediments of standard curve analysis include i) the requirement to obtain standards for each GOI; ii) the need to generate a separate standard curve for each GOI for each qPCR run; iii) the efficiency obtained from the standard curve may not be representative of the samples used in experiments; and iv) cost and time involved [24]. Thus, standard curves are laborious, whereas a single AccuCal calibration curve, and analysis by RealCount, can be used to quantify all GOIs on the same PCR plate, thereby making the AccuCal method simpler, cheaper and quicker to perform.

PCR efficiencies are rarely perfect and they may vary between samples even under identical reaction conditions, e.g. due to slight differences in the quantity of inhibitors present [25]. A small change in efficiency from 2.00 to 1.97 over 30 cycles equates to a 57 % difference in input DNA calculation, while a change from 2.00 to 1.90 over the same range makes 365 % difference (calculated using ((2^n^/E^n^)-1) × 100, where E is efficiency and n is cycle number). Methods that assume perfect efficiency (i.e. 2) such as ΔΔCq analysis [11] are thus flawed [26]. The ‘Pfaffl’ method [12] of analysis is more accurate as it uses the efficiencies of the individual primers. However, these efficiencies are onerous to obtain, requiring a standard curve to be generated for each GOI. Over the last 10–15 years, many researchers have made progress in developing algorithms for single sample kinetic PCR analysis [10, 27–32]. We have selected one of these [10] for incorporation into RealCount software to automate individual efficiency calculations and therefore improve upon the accuracy of the quantification over traditional methods which use an averaged or assumed efficiency.

## Conclusion

In summary, we have shown that AccuCal provides an easy alternative to traditional qPCR quantification methods. AccuCal removes the bias of troublesome reference genes and provides the accuracy of standard curves without the hassle. It can be used in every qPCR experiment to standardize dye- or probe-based assays. RealCount software automates the quantification process. The simplicity of AccuCal, its broad utility and the ability to quantify all GOIs on a PCR plate, make AccuCal a truly universal calibrator.

## Methods

### AccuCal calibrators

AccuCal-D (Accugen) is a proprietary 90 bp, 44 % GC content, double-stranded DNA calibrator that can be used to provide an accurate, absolute quantification in qPCR using intercalating dyes. AccuCal-P (Accugen) comprises a range of proprietary 21 bp, 62 % GC content, single-stranded DNA calibrators that are labelled on the 5′ end by conventional means with any single fluorophore molecule per moiety and can be used for absolute quantification in probe-based qPCRs. In these experiments, AccuCal-P was labelled with FAM, but any fluorophore that matches the excitation and emission spectra of the fluorophore on the detection probe for the gene(s) of interest (GOIs) can be used. Multiple Accual-P calibrators labelled with different fluorophores can also be added simultaneously to the same well to enable multiplexing if desired. The methodology for using AccuCal-D and AccuCal-P, collectively referred to as AccuCal, is the same.

### Generating an AccuCal calibration curve

We performed an initial calibration run on each qPCR platform using a range of dilutions of AccuCal-D or AccuCal-P as appropriate, from 0 ng up to 500 ng per well to determine the optimal range of AccuCal to use. AccuCal was diluted in nuclease-free water (Sigma) and made up in the same PCR master mix, and to the same volume, as used during PCR, omitting primers and template. The calibrator, although not amplified, was subjected to PCR cycling under the conditions used for amplifying the GOIs, and the fluorescence data acquired at the end of each cycle as normal. We then determined the optimal range of AccuCal to use by importing the raw fluorescence data into RealCount software (Accugen), subtracting the background fluorescence reading, which is provided by the 0 ng AccuCal (comprising water and master mix only), and choosing those concentrations that produce a linear calibration curve within the detection limits of the platform in question. Typically, the ideal range is between 0 ng and 200 ng and should span the detectable exponential portion of the amplification curve between the take-off point and the second derivative maxima. At least six concentrations of AccuCal spanning the optimal range and including a 0 ng control is then used on every subsequent PCR, preferably in duplicate, to produce a calibration curve.

### Quantification using AccuCal and RealCount

Following qPCR amplification of the GOIs, and inclusion of the pre-determined range of AccuCal calibrators, the raw fluorescence data was imported into RealCount software and the calibration curve automatically plotted. The software calculates the efficiency of individual qPCR amplifications over the exponential portion of the amplification curve using a published algorithm [10]. From the calibration curve, we determined the pmols of DNA over the exponential portion of each amplification curve and calculated the mean initial input DNA using the equation pmz = pm/E^n^, where pmz is pmols at time zero, E is efficiency and n is cycle number. The pmz is converted into copies/PCR using pmz × 6.022 × 10^23^ × 10^−12^. The software provides a mean, standard deviation and standard error of the mean output for the quantification of the initial input amount of the gene(s) of interest in the qPCR reaction during the entire detectable exponential phase.

### Tissue culture, animal handling, mouse models and tissue harvesting

We seeded 10^5^ human peripheral blood mononuclear cells (PBMCs) in culture medium (X-Vivo 15 (Lonza) containing 10 mM HEPES (Life Technologies), 2 mM L-glutamine (Life Technologies) with 50 μM 2-mercaptoethanol (Amresco)) per well in a 96-well round bottom plate in quadruplicate and incubated at 37 °C with 5 % CO_2_ for 24 h with either culture medium alone, 0.25x phorbol myristate acetate (PMA)/ionomycin (5 ng mL^-1^ PMA (Sigma) and 125 ng mL^-1^ ionomycin (Sigma)), 0.5x PMA/ionomycin (10 ng mL^-1^ PMA, 250 ng mL^-1^ ionomycin) or 1x PMA/ionomycin (20 ng mL^-1^ PMA, 500 ng mL^-1^ ionomycin). Cells were washed in PBS and either harvested directly for flow cytometry or the cell pellet was suspended in 100 μl Cells-to-Signal lysis buffer (Life Technologies) for RNA isolation.

Mice were housed on a 12 h:12 h cycle of light and dark at 19–22 °C. Mice were fed food and water *ad libitum*. All experiments were approved by the Sydney South West Area Health Service Animal Welfare Committee within National Health and Medical Research Council guidelines for animal experimentation. The prostate epithelium specific phosphatase and tensin homolog knockout (pePTENKO; FVB/N background) mice were generated by crossing Pten^loxp/loxp^ mice [33] to the probasin Cre (Tg(Pbsn-cre)) transgenic line [34]. In Tg(Pbsn-cre) mice the Cre is controlled by the prostate epithelial cell-specific, modified probasin gene promoter. Cre negative littermates were used as wild-type controls (denoted as WT).

We genotyped experimental mice using genomic DNA from mouse tails amplified by PCR. The primer pair sequences used for genotyping mice were as follows: Cre forward 5′-CTGACCGTACACCAAAATTT GCCTG-3′ and reverse 5′-GATAATCGCGAACATCTTCAGGTTC-3′ [35], PTEN forward 5′- TCCCAGAG TTCATACCAGGA -3′ and PTEN reverse-1 5′- GCAATGGCCAGTACTAGTGAAC -3, PTEN reverse-2 5′- AATCTGTGCATGAAGGGAAC -3′ [36].

Anterior prostate was collected from WT and pePTENKO male mice at average age of 20 weeks. Mice were anesthetized with ketamine/xylazine and euthanized by cardiac exsanguination. Anterior prostate was dissected free of surrounding fat, snap frozen in liquid nitrogen and stored at −80 °C for further RNA or protein extraction.

### Ethics statement

Human PBMCs were collected from healthy donors with informed written consent following approval by the Western Sydney Local Health District (WSLHD) Human Research Ethics Committee under permit number HREC 2002/9/3.6(1425). The consent forms are vetted and approved by the Scientific Advisory Committee and the WSLHD Human Research Ethics Committee, and are then securely stored as a hard copy for 15 years under current NH&MRC requirements.

All animal experiments were approved by the Sydney South West Area Health Service Animal Welfare Committee within National Health and Medical Research Council guidelines for animal experimentation (permit number 2009/009B). All efforts were made to minimize suffering.

### Isolation of RNA and cDNA synthesis

Following stimulation with varying concentrations of PMA and ionomycin, we isolated RNA from 100,000 human PBMCs using the RNeasy mini kit (Qiagen) as per the manufacturer’s instructions. RNA was reverse transcribed using qScript cDNA supermix (Quanta Biosciences) according to the manufacturer’s instructions. We checked the concentration, purity and integrity of RNA using a Bioanalyzer (Illumina).

RNA was extracted from approximately 30 mg of anterior prostate from WT and pePTENKO mice using the RNeasy Mini kit (Qiagen) according to the manufacturer’s instructions. The concentration and purity of total RNA was measured using the Nanophotometer (Implen) by measuring absorbance at 260, 280 and 230 nm. RNA integrity was assessed by gel electrophoresis. To remove residual genomic DNA contamination, 2 μg of RNA was treated with ribonuclease-free DNase I (0.5 U μg^-1^ RNA; Invitrogen). 1 μg of RNA was reverse transcribed to produce cDNA utilizing the Superscript III first strand synthesis SuperMix for qRT-PCR kit (Invitrogen) according to the manufacturer’s protocol.

### Real-time PCR

We undertook amplification of a ten-fold dilution series of lambda DNA (*Hin*d III digest, New England Biolabs), ranging from 4.5 × 10^6^ – 4.5 × 10^1^ and quantified using a NanoDrop 1000 spectrophotometer (Thermo Scientific), in Sso Fast EvaGreen Supermix (Bio-Rad) as per manufacturer’s instructions, in a 20 μl final volume. The expected amplicon size is 92 bp and the primer sequences are listed in Table 1. PCR cycling was performed at 95 °C, 2 min, followed by 40 cycles of 95 °C, 5 s; 60 °C, 30 s on a RotorGene-6000 (Qiagen), with the fluorescence acquired at the end of each cycle and the gain set to 7 in the green channel. A melt curve performed at the end of the amplification was undertaken to confirm that there is only a single product amplified in each reaction.

**Table 1:** Primers used for qPCR studies

Gene	Accession number	Primer sequence	Annealing temperature	Amplicon size
Lambda	NC_001416	For: CGGCGTCAAAAAGAACTTCC	60 °C	92 bp
		Rev: GCATCCTGAATGCAGCCATA		
Lambda	NC_001416	For: CGGCGTCAAAAAGAACTTCC	60 °C	501 bp
		Rev: TGATCCCACCTCATTTTCATGT		
*Ar*	NM_013476.3	For: ACCCAAAACCCACCTTGTT	64 °C	214 bp
		Rev: ACGCAGCAGATTCAAAATGT		
*Actb*	NM_007393.3	For: AGCCATGTACGTAGCCATCC	64 °C	377 bp
		Rev: GGAACCGCTCGTTGCCAATA		
*Rpl19*	NM_009078.2	For: GATCATCCGCAAGCCTGTGACT	60 °C	362 bp
		Rev: GTGCTTCCTTGGTCTTAGAC		
*Hmbs*	NM_001110251.1	For: TGATGAAAGATGGGCAACTG	64 °C	160 bp
		Rev: ATGTTACGGGCAGTGATTCC		
*Rps9*	NM_029767.2	For: ATTACATCCTGGGGCCTGAAG	64 °C	210 bp
		Rev: AAGGAGAACGGAGGGAGAAG		
*Ppia*	BC083076.1	For: ATCACGGCCGATGACGAGCC	64 °C	217 bp
		Rev: TCTCTCCGTAGATGGACCTGC		
*Hprt*	NM_013556.2	For: GATACAGGCCAGACTTTGTTGG	64 °C	154 bp
		Rev: AACTTGCGCTCATCTTAGGC		
*CD40*	NG_007279	For: GAAACTGGTGAGTGACTGC	60 °C	341 bp
		Rev: CACATTGGAGAAGAAGCC		
*IL7R*	NG_009567	For: CTGGAACATCTTTGTAAGAAACCAAG	60 °C	127 bp
		Rev: TAGCTTGAATGTCATCCACCCT		
*GAPDH*	NG_007073.2	For: TCCACCACCCTGTTGCTGTA	60 °C	231 bp
		Rev: ACCACAGTCCATGCCATCAC		

Serial dilutions of lambda DNA were also amplified as above in either Sso Fast EvaGreen Supermix or in Go Taq hot start colorless mastermix (Promega), the latter being detected with an hydrolysis probe specific to the amplicon and having the sequence 5′ 56-FAM/aacactcaggcacgcggtctg/3IABkFQ 3′ (Integrated DNA Technologies). Suitable amounts of both AccuCal-D and AccuCal-P (between 0 ng and 200 ng for both) were run alongside the PCRs and all PCRs were quantified using the calibration curves generated by both the calibrators independently.

Lambda amplicons sized 92 bp and 501 bp, which span the typical range for real-time qPCR, were amplified using the primers listed in Table 1 and the same cycling conditions as above with the exception of an annealing/extension time of 1 min for the larger product.

Researchers in eight independent labs tested the universality and accuracy of AccuCal-D on various qPCR machines and in different mastermixes. The qPCR platform/mastermix combinations tested were RotorGene 6000 (Qiagen)/SensiMix™ SYBR® Hi-Rox (Bioline), Mastercycler® ep series (Eppendorf)/GoTaq® qPCR mastermix (Promega), CFX™96 (Biorad)/Sso Advanced™ SYBR® green (Bio-Rad), ViiA™7 #1 (Life Technologies)/GoTaq® qPCR mastermix (Promega), ViiA™7 (#2)/Fast SYBR® green (Life Technologies), 7900HT (Applied Biosystems)/EXPRESS SYBR® GreenER™ qPCR supermix universal (Life Technologies), Eco (Illumina)/Power SYBR® green (Applied Biosystems) and qTOWER (Analytik Jena)/Sso Fast™ EvaGreen® (Bio-Rad). In all cases, in addition to the researchers’ own PCR samples, AccuCal-D was included on the same PCR plate alongside a set of ten-fold serial dilutions of lambda (4.5 × 10^5 ^ – 4.5 × 10^1^, plus no template control) which were amplified to examine the accuracy of the AccuCal quantification on each of these platforms and in each of these mastermixes.

For PBMC experiments, qPCR was conducted in Power SYBR Green master mix (Life Technologies) in a 12 μl reaction volume on an Eco real time PCR machine (Illumina) using the cDNA generated from 100,000 cells in each reaction. AccuCal-D was included at 0 ng, 10 ng, 20 ng, 30 ng, 50 ng, 70 ng and 100 ng per plate. The primers used to amplify *CD40,* interleukin 7 receptor α chain (*IL7R*, also known as *CD127*) and glyceraldehyde 3-phosphate dehydrogenase (*GAPDH*) are listed in Table 1 and the cycling conditions were 96 °C, 10 min; 5 cycles (95 °C, 30 s; 64 °C, 30 s; 72 °C, 30 s); 40 cycles (95 °C, 30 s; 60 °C, 30 s; 72 °C, 30 s); followed by a melt curve analysis. We analyzed the results by absolute quantification using AccuCal and RealCount, or traditional relative quantification using ΔΔCq [11] or Pfaffl [12] analyses with *GAPDH* as the reference gene and no PMA/ionomycin as the control. For Pfaffl analysis, the amplification efficiency values generated by RealCount were used. The absolute values were also expressed relatively by calculating the absolute quantification in stimulated cells/absolute quantification in cells with no PMA and ionomycin. For relative quantification methods, the control samples (No PMA/ionomycin in this case) always have a value of 1.

The expression levels of androgen receptor (*Ar*) and the reference genes beta-actin (*Actb*), ribosomal protein S9 (*Rps9*)*,* hypoxanthine guanine phosphoribosyl transferase (*Hprt*), peptidylprolyl isomerase A (*Ppia,* also known as cyclophilin), ribosomal protein L19 (*Rpl19*) and hydroxymethylbilane synthase (*Hmbs*)*,* in anterior prostate from both WT and pePTENKO mice were assessed by qPCR in either the CFX connect real time PCR detection system (Bio-Rad) or RotorGene-6000 (Qiagen). The reactions were performed in duplicate using the SensiMix™ SYBR® Hi-Rox kit (Bioline) in a 10 μl reaction volume with the cDNA reverse transcribed from 20 ng total RNA per reaction, according to manufacturer’s instructions. The primers used are listed in Table 1 and the cycling conditions comprised 95 °C for 10 min; 45 cycles (95 °C, 30 s; 64–67 °C, 20 s; 72 °C, 30 s); 72 °C, 2 min. AccuCal-D was included on the plate at 0 ng, 10 ng, 20 ng, 30 ng, 40 ng and 50 ng in duplicate. A melt curve analysis was performed at the end of the reaction in order to check for primer-dimer formation and contamination. The absolute quantification results were acquired by using AccuCal-D and RealCount and, for *Ar* and *Actb*, standard curves were also generated by amplifying serial dilutions of separate plasmids containing the amplicons. Relative quantification was undertaken by normalizing the *Ar* with either *Actb* or *Hmbs* as the reference gene and WT as the control for ΔΔCq analysis [11] or Pfaffl analysis [12]. The absolute values were also expressed relatively by calculating the absolute quantification in pePTENKO/absolute quantification in WT.

For all qPCRs, statistical differences between the samples and control were examined by unpaired, two-sample, Student’s *t*- test. Analysis of the slopes were also undertaken to determine differences in the quantification observed between the standard curves and AccuCal/RealCount.

### Flow cytometry

The levels of CD40 and IL7R α chain on the surface of the cells were also measured by flow cytometry. Following stimulation, we transferred cells to flow cytometry tubes and incubated with 2.5 μl of Peridinin-Chlorophyll-protein conjugated antibody to CD45 (Biolegend, 304002, HI30 clone), 2.5 μl of Allophycocyanin conjugated antibody to CD40 (eBioscience, 17-0409-42, Clone 5C3) and 2.5 μl Phycoerythrin conjugated antibody to IL7R α chain (eBioscience, 12-1271-42, clone eBioRDR5) for 30 min on ice. Cells were washed once with 3 mL chilled PBS containing 0.05 % sodium azide and then suspended in 250 μl of the same buffer and read on a FACSCanto II instrument (BD Biosciences). FACSDiva fcs files were analyzed by FloJo Software (Tree Star Inc.). In all cases, CD45+ cells were gated and these gated cells were prepared as histograms before analysis to determine median values for CD40 and IL7R.

### Western analysis

The level of androgen receptor (AR) and beta-actin (ACTB) protein was assessed in anterior prostate tissue from WT and pePTENKO mice by Western blot analysis using standard techniques. In brief, anterior prostate tissue was lysed in ice-cold RIPA lysis buffer (50 mM Tris, 150 mM NaCl, 0.5 % Deoxycholate, 0.1 % SDS, 1 % v/v Triton X-100, Complete Protease Inhibitor tablet [Roche], phosphatase inhibitor tablet [PhosSTOP, Roche], pH 7.8). We homogenized the tissue in Lysing Matrix S tubes with metal beads (MP Biomedicals) using the PowerLyzer (MO-BIO laboratories Inc) at 2,000 rpm for 15 s. The lysates were incubated for 30 min at 4 °C and then centrifuged at 13,400 *g* for 15 min. Supernatant was retained and transferred to a clean tube. Protein concentrations were determined by Micro BCA assay (Pierce) as per manufacturer’s instructions. Equal amounts of protein (20 μg) were subjected to electrophoresis on ready-made NuPage 10 % Bis-Tris gels (Invitrogen) and then transferred electrophoretically onto a 0.45 μm Hybond ECL nitrocellulose membrane (GE Healthcare). The membrane was blocked overnight at 4 °C with 5 % skim milk in TBST (20 mM Tris, 137 mM NaCl, 0.05 % Tween-20) and then blotted overnight at 4 °C with antibody to AR (1:1,000 in 5 % skim milk-TBST; N-20, sc-816; Santa Cruz Biotechnology, Inc.) or ACTB (1:1,000 in 5 % skim milk-TBST; 4967, Cell Signaling Technology). Blots were washed with TBST (3 × 10 min) and probed with donkey anti-rabbit IgG horseradish-peroxidase conjugated antibody (1:2,000; GE Healthcare) followed by another round of 3 × 10 min TBST washes. Bands were developed using the ECL western blotting detection kit (GE Healthcare) as per manufacturer’s instructions on a ChemiDox XRS System (Bio-Rad Laboratories). The membranes were analyzed using Quantity One Software (Bio-Rad Laboratories) and images captured on the chemiluminescence setting, exposure times varied according to the band intensity. The signal volumes for each band were measured with Quantity One Software in order to quantify the amount of protein present. Statistical differences between the samples and control were examined by unpaired, two-sample, Student’s *t*- test.

### Immunohistochemistry analysis

In order to determine protein distribution in tissues, we fixed anterior prostate from WT and pePTENKO mice in 4 % paraformaldehyde in PBS (137 mM NaCl, 2.7 mM KCl, 10 mM Na_2_HPO_4_, 2 mM KH_2_PO_4_) overnight at 4 °C and transferred to 70 % ethanol. Tissues were blocked in paraffin and immunohistochemistry for AR (1:100 in PBS containing 10 % Pierce Superblock (Thermo Fisher Scientific); N-20, sc-816; Santa Cruz Biotechnology Inc.) was performed on 5 μm thick dewaxed paraffin sections. Microwave-induced antigen retrieval was done with 0.01 M citrate buffer (648 mL of 0.1 M sodium citrate, 152 mL of 0.1 M citric acid (pH 6.0)) for 15 min. Sections were washed in PBS, blocked for 30 min with 3 % H_2_O_2_ in methanol followed by 1 h with Pierce Superblock containing 0.5 % bovine serum albumin and incubated 1 h at room temperature with primary antibodies, followed by three washes in PBS. We visualized the signal with Vectastain Elite ABC Kit containing biotinylated anti-rabbit secondary antibody (Vector Laboratories) followed by color development with 3,3′-diaminobenzidine tetrahydrochloride chromogenic substrate (Dako). Sections were counterstained with Harris hematoxylin and coverslipped for microscopy. Photos of immunostained prostate sections were acquired using an EVOS FL Auto Imaging System (Life Technologies) with a 10× objective.

## Additional file

Additional file 1: Supporting data for Figs 1-4. (XLS 603 kb)

## Abbreviations

ACTB: β-actin
AR: androgen receptor
bp: base pairs
Cq: quantification cycle
GOIs: genes of interest
*Hmbs*: hydroxymethylbilane synthase
*Hprt*: hypoxanthine guanine phosphoribosyl transferase
MIQE: Minimum Information for publication of Quantitative real-time PCR Experiments
PBMCs: peripheral blood mononuclear cells
pePTENKO: Prostate epithelium-specific phosphatase and tensin homolog knockout
PMA: phorbol myristate acetate
*Ppia*: peptidylprolyl isomerase A
qPCR: quantitative real-time PCR
*Rpl19*: ribosomal protein L19
*Rps9*: ribosomal protein S9
RT: reverse transcription
WSLHD: Western Sydney Local Health District
WT: wild-type
